# Occurrence of Yam Mosaic Virus and Yam Mild Mosaic Virus on *Dioscorea* spp. Germplasm Collection in Cuba—Epidemiology of Associated Diseases

**DOI:** 10.3390/plants13182597

**Published:** 2024-09-17

**Authors:** José Efraín González Ramírez, Dariel Cabrera Mederos, Vaniert Ventura Chávez, Rosa Elena González Vázquez, Katia Ojito-Ramos, Liset García Romero, Luis Fabián Salazar-Garcés, Diana Catalina Velastegui-Hernández, Elena Vicenta Hernández Navarro, Michel Leiva-Mora, Fabián Giolitti, Orelvis Portal

**Affiliations:** 1 Instituto de Investigaciones de Viandas Tropicales, Santo Domingo 53 000, Cuba; vaniertvc1983@gmail.com (V.V.C.); rosaelena880223gonzalez.vazquez@gmail.com (R.E.G.V.); 2 Unidad de Fitopatología y Modelización Agrícola (CONICET-INTA), Consejo Nacional de Investigaciones Científicas y Técnicas, Av. 11 de Septiembre 4755, Córdoba X5020ICA, Argentina; cabrera.dariel@inta.gob.ar (D.C.M.); giolitti.fabian@inta.gob.ar (F.G.); 3 Instituto de Patología Vegetal, Centro de Investigaciones Agropecuarias, Instituto Nacional de Tecnología Agropecuaria, Av. 11 de Septiembre 4755, Córdoba X5020ICA, Argentina; 4 Departamento de Biología, Facultad de Ciencias Agropecuarias, Universidad Central “Marta Abreu” de Las Villas, Carretera a Camajuaní km 5.5, Santa Clara 54 830, Cuba; kojito@uclv.edu.cu (K.O.-R.); lisetgr9999@gmail.com (L.G.R.); 5 Facultad de Ciencias de la Salud, Universidad Técnica de Ambato, Ambato 180207, Ecuador; lf.salazar@uta.edu.ec (L.F.S.-G.); dc.velastegui@uta.edu.ec (D.C.V.-H.); ev.hernandez@uta.edu.ec (E.V.H.N.); 6 Laboratorio de Biotecnología, Facultad de Ciencias Agropecuarias, Universidad Técnica de Ambato (UTA-DIDE), Cantón Ceballos vía Quero, Sector El Tambo-La Universidad, Ceballos 1801334, Ecuador; m.leiva@uta.edu.ec; 7 Centro de Investigaciones Agropecuarias, Facultad de Ciencias Agropecuarias, Universidad Central “Marta Abreu” de Las Villas, Carretera a Camajuaní km 5.5, Santa Clara 54 830, Cuba

**Keywords:** potyvirus, yams, virome

## Abstract

Potyvirus diseases are one of the main challenges facing the production of yam (*Dioscorea* spp.). The objective of this study was to identify the potyviruses present in the *Dioscorea* spp. germplasm collection at Instituto de Investigaciones de Viandas Tropicales (INIVIT) to establish methodologies for the characterization of the associated diseases. For this purpose, immunochemical and molecular methods were used to identify the potyviruses present. The symptomatology of *Dioscorea* spp. at INIVIT’s germplasm collection was described. In addition, the severity and incidence in the germplasm collection and production areas were evaluated. As a result, the first report of yam mosaic virus (*Potyvirus yamtesselati*) and yam mild mosaic virus (*Potyvirus yamplacidum*) in Cuba is presented. The existence of resistant, tolerant, and susceptible cultivars to potyvirus-associated diseases in the germplasm collection was detected, and the incidence of these diseases was higher than 64% in the production areas evaluated. This study represents a step forward in the establishment of certification programs for propagating material of *Dioscorea* spp. in Cuba.

## 1. Introduction

The genus *Dioscorea* L. comprises more than 600 species [1,2]. Around 60 species of this wide diversity serve as food and medicinal plants [3], and 10 are cultivated for commercial purposes [4]. One of the major constraints facing the production of yam (*Dioscorea* spp.) at the global level is the impact of viral diseases affecting the production areas [5]. Badnaviruses are the most prevalent [6]; however, potyviruses have the greatest economic impact [7,8] and are considered to cause agricultural yield losses of more than 50% [9].

In Cuba, different species are distributed in the central and eastern regions, where the most widespread cultivars belong to the species *Dioscorea alata* L. and *Dioscorea cayenensis* subsp. *rotundata* (Poir) J. Miège. The highest production volumes are achieved in the provinces of Guantánamo and Villa Clara [10].

The Instituto de Investigaciones de Viandas Tropicales (INIVIT) has been assigned by the Ministry of Agriculture (MINAG) of Cuba to lead the development of roots and tubers, such as *Dioscorea* spp. In this institution, there is a *Dioscorea* spp. germplasm collection with approximately 120 accessions belonging to five species, i.e., *D. alata*, *D. cayenensis*, *Dioscorea bulbifera* L., *Dioscorea esculenta* (Loir.) Burkill and *Dioscorea trifida* L. f. [11]. INIVIT is the main supplier of propagation material for *Dioscorea* spp. (in vitro and agamic-produced plants) in the country [10]. Due to the recent identification of potyviruses in experimental areas, INIVIT has established a potyvirus-free seed production program [12], although without identification of the viruses present. On the other hand, measures for the management of associated diseases under field conditions have been established [13].

This study reports the evaluation of the symptomatology, identification of the potyvirus present, prevalence and diversity in the *Dioscorea* spp. at INIVIT’s germplasm collection and in commercial farms in the municipality of Camajuaní, Cuba.

## 2. Results

### 2.1. Symptom Classification

**Figure 1:**
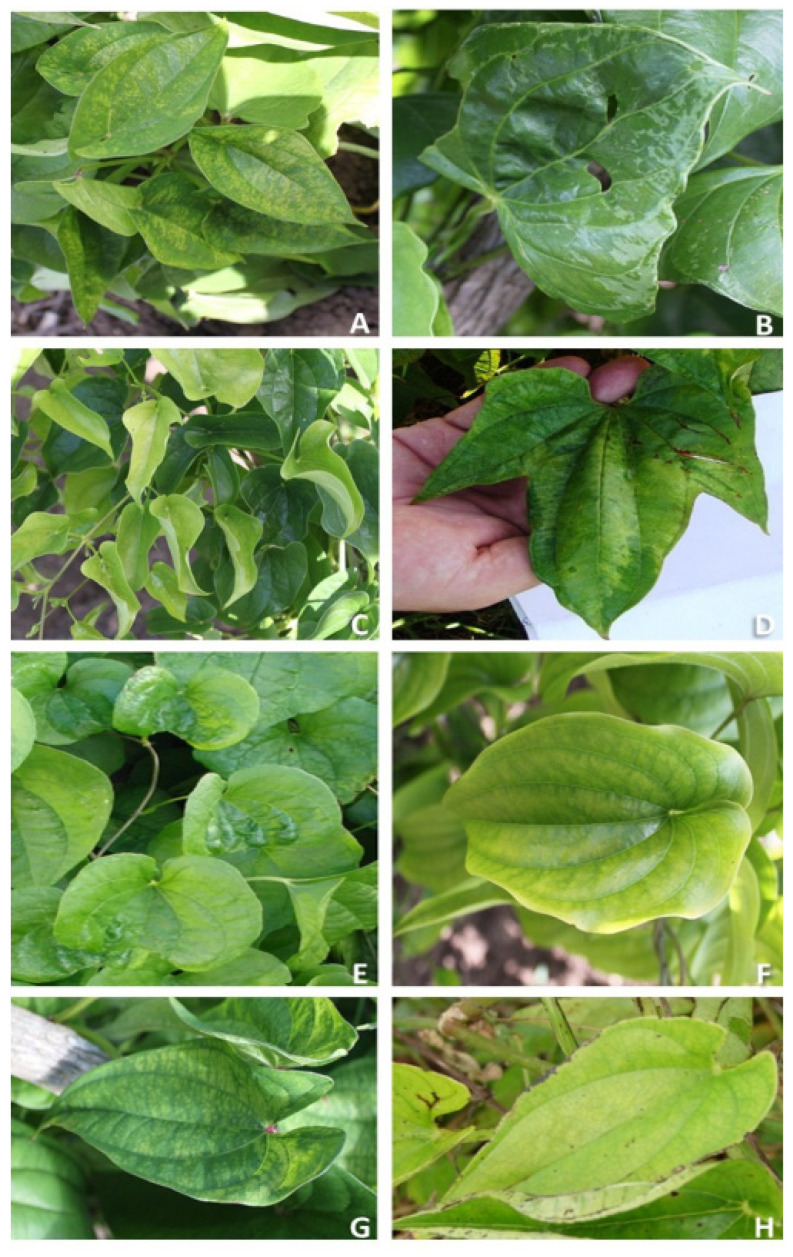
Frequent symptoms observed in leaves of *Dioscorea* spp. plants at INIVIT’s germplasm collection. (**A**) yellow banding in *D. alata* (YB-a); (**B**) mild mosaic in *D. alata* (MM-a); (**C**) curling and chlorosis in *D. cayenensis* subsp. *rotundata* (CC-r); (**D**) severe mosaic in *D. trifida* (SM-t); (**E**) green swelling zones in *D. esculenta* (GSZ-e); (**F**) mild mosaic in *D. cayenensis* subsp. *cayenensis* (MM-c); (**G**) yellow banding in *D. cayenensis* subsp. *cayenensis* (YB-c); (**H**) severe chlorosis in *D. bulbifera* (SC-b).

A total of 479 symptomatic leaf samples were collected from the *Dioscorea* spp. germplasm collection. They were classified into seven groups according to the symptoms, i.e., severe mosaic (SM), curling and chlorosis (CC), yellow banding (YB), mild mosaic (MM), green swelling zones (GSZ), severe chlorosis (SC) and leaf distortion (LD) (Figure 1). Also, 120 asymptomatic leaves were collected (50 from *D. alata*, 40 from *D. cayenensis* subsp. *rotundata*, 10 from *D. cayenensis* subsp. *cayenensis*, 10 from *D. esculenta* and 10 from *D. bulbifera*), and none from *D. trifida*.

The total number of leaf samples collected was classified into each of the 25 symptom-species groups (Table 1). In all *Dioscorea* species sampled, at least three of the described symptoms were observed, although in no case was it possible to establish a sequence between them. Nor was the presence of two symptoms detected simultaneously on the same plant, but within each plot (16 plants of the same cultivar) in which the *Dioscorea* spp. germplasm collection is divided.

CC symptom was manifested along sections of plant vines in *D. cayenensis* subsp. *rotundata* (Figure 1C), while SM symptom was observed on the whole leaves, occupying the entire vines in *D. trifida* (Figure 1D). The remaining symptoms were observed in less numerous groups of leaves.

CC symptom was the most frequent symptom in *D. cayenensis* subsp. *rotundata* with 55%, and was only detected in this subspecies. YB and MM symptoms were the most frequent in all samplings (106 and 62, respectively). The presence of both symptoms was higher in *D. alata*, where they were recorded at 79% and 73%, respectively. In this study, SM symptoms were only recorded in *D. trifida* and the two subspecies of *D. cayenesis*.

In some of the *Dioscorea* species sampled, a single symptom predominated. In this regard, SM symptoms characterized 79% of the samples collected in *D. trifida*. On the other hand, CC symptom was observed in 82% of the samples collected in *D. bulbifera*.

In *D. alata* and *D. cayenensis* subsp. *rotundata*, the variability of symptoms in the collected samples was higher, up to five symptoms. In *D. esculenta*, GSZ symptoms had the highest frequency of occurrence. In addition to *D. esculenta*, this symptom was found only in *D. alata* samples.

The monitoring of symptom development in the marked plants allowed the establishment of different patterns for some of the established symptom-species groups. MM-a (in *D. alata*) and MM-r (in *D. cayenensis* subp. *rotundata*) symptoms were observed for the first time, masked in some cases without occupying the plant vines. The same occurred with YB-c (in *D. cayenensis* subp. *cayenensis*), GSZ-r, MM-c symptoms, which sometimes did not manifest themselves after the first evaluation.

The observations were made during the phenological development of *Dioscorea* spp. from April 2015 to February 2018. During this period, the mean monthly maximum temperature exceeded 30 °C nine times in 2015, six times in 2016, and five times in 2017 (Supplementary Material Table S1).

**Figure 2:**
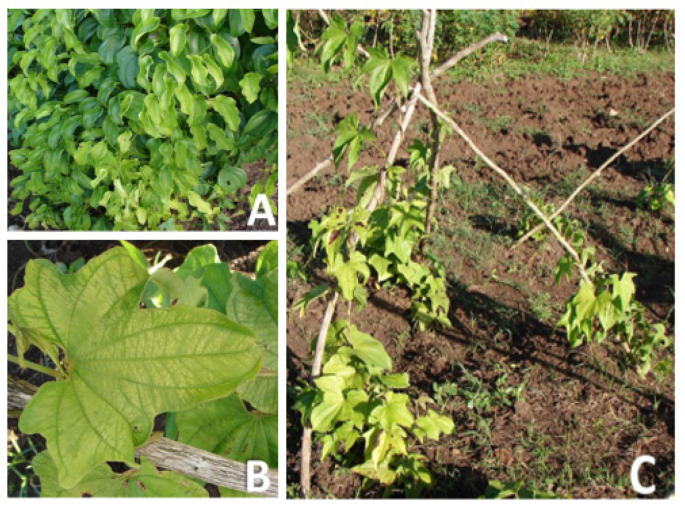
Evolution of symptoms in *Dioscorea* spp. plants. (**A**) CC symptom along sections of the vides in *D. cayenensis* subp. *rotundata*); (**B**) SM symptom occupying the entire leaf blade in *D. trifida*; (**C**) SM symptom occupying the entire plant vine in *D. trifida*.

The CC-r symptom was always present in the plants where it was observed and occupied sections in the vines (Figure 2A). However, with subsequent growth of the vines, asymptomatic leaves were found in the youngest sections. SM-t (in *D. trifida*) symptom was the most persistent of all. After its appearance, it was observed to develop until it occupied almost the entire leaf area of the plant (Figure 2B,C).

### 2.2. Potyvirus Detection

#### 2.2.1. Electron Microscopy

**Figure 3:**
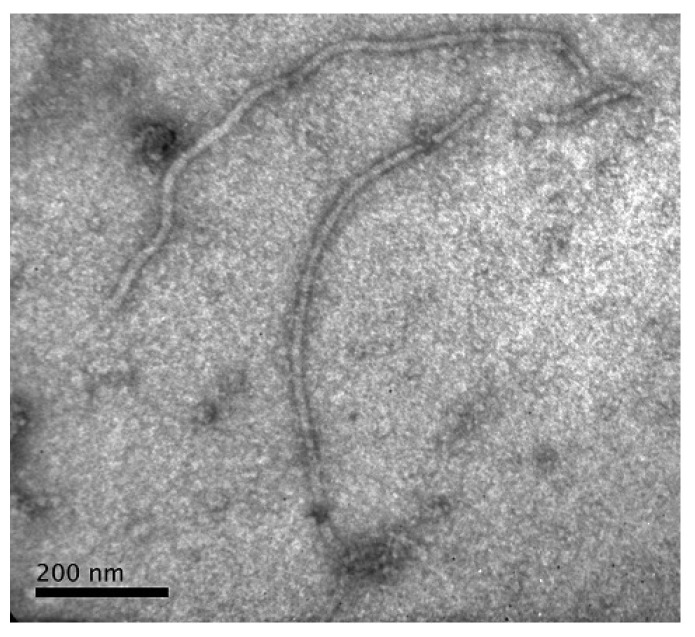
Flexuous and filamentous particles typical of the genus Potyvirus (scale bar = 200 nm) observed by electron microscopy in leaf samples collected from *Dioscorea* spp.

The particles observed were similar in morphology and size to those described for potyviruses. In leaf samples collected with SM, CC, YB, MM, and GSZ symptoms, regardless of plant species, flexible particles between 650 and 950 nm long were found, which are consistent with those described for the genus *Potyvirus* (Figure 3). No particles were observed in asymptomatic samples.

#### 2.2.2. ELISA

Of the 25 symptom-species groups, 14 were positive for potyvirus by ACP-ELISA (Table 2). In all assays performed, the cut-off was determined to be between 0.2 and 0.22 absorbance value (AV). In potyvirus-positive samples, the AV ranged from the cut-off up to 1.2. Positive reactions were obtained in all samples of *Dioscorea* spp. analyzed, except in those showing SC and LD symptoms. Asymptomatic samples were negative.

The AV allows the quantitative establishment of different reactions between the determined symptom-species groups. In this study, AV in samples with YB-a, MM-a, and CC-r symptoms ranged from 0.42 to 0.6, while the SM-t symptom was higher than 0.85, corresponding to two and four times the cut-off limit, respectively.

The ACP-ELISA analyses of the symptomatic samples showed that *D. trifida* is the most susceptible species to the potyviruses present in the experimental conditions, while *D. alata* and *D. cayenensis* subsp. *rotundata* show an intermediate response between tolerance and susceptibility.

#### 2.2.3. RT-PCR

In RT-PCR with NIb2F/NIb3R primers, amplification of the expected band size (~350 bp) was obtained in 12 of the 14 symptomatic species groups evaluated (Table 3). Amplification was not obtained in the asymptomatic samples, confirming the results obtained by ACP-ELISA. Amplification was also not obtained in the YB-b (in *D. bulbifera*) and MM-b symptomatic samples. In both cases, it is a single sample (Table 1) collected in *D. bulbifera*.

In six of the 14 symptom-species groups evaluated, amplification was obtained with a band size of ~586 bp, which is expected for YMV detection (Table 3). The presence of YMV was detected in samples with YB-a, MM-a, CC-r, SM-r, SM-t, and YB-c symptoms, which include three of the species conserved in the *Dioscorea* spp. at INIVIT’s germplasm collection, except *D. esculenta* and *D. bulbifera*.

Similarly, YMMV was detected in seven of the 14 symptom-species groups studied, as confirmed by RT-PCR amplification of a ~249 bp fragment (Table 3). The presence of YMMV was detected in samples with YB-a, MM-a, SM-r, MM-r, MM-r, SM-c, MM-c, and GSZ-e (in *D. esculenta*) symptoms, which included three of the *Dioscorea* species conserved at INIVIT. YMMV has not been detected in *D. bulbifera*.

All samples positive for YMV or YMMV by RT-PCR were also classified as potyvirus positive by ACP-ELISA. Neither YMV nor YMMV was detected in the 28 samples of *D. bulbifera* collected and diagnosed from symptomatic leaves during the three-year period.

On the other hand, three of the symptom-species groups described (YB-a, MM-a, and SM-r) tested positive for both potyviruses, indicating the presence of mixed YMV and YMMV infections in the samples evaluated.

The detection and characterization of two potyviruses in more than 120 accessions of the five *Dioscorea* species from Africa, Asia, Oceania, and the Americas conserved at INIVIT’s germplasm collection has established a gradient of interactions between plants and these viruses. The Cuban cultivars of *D. trifida*, a species of American origin [1] evaluated in this study, do not complete their cycle. The vines emitted by the plants die from 180 to 210 days after planting (dap), and the tubers do not reach commercial value. In the experimental conditions of cultivation of *D. alata* and *D. cayenensis*, they were affected by YMV and YMMV. However, the cultivars evaluated managed to complete the crop cycle.

#### 2.2.4. Sequencing and Analysis

**Figure 4:**
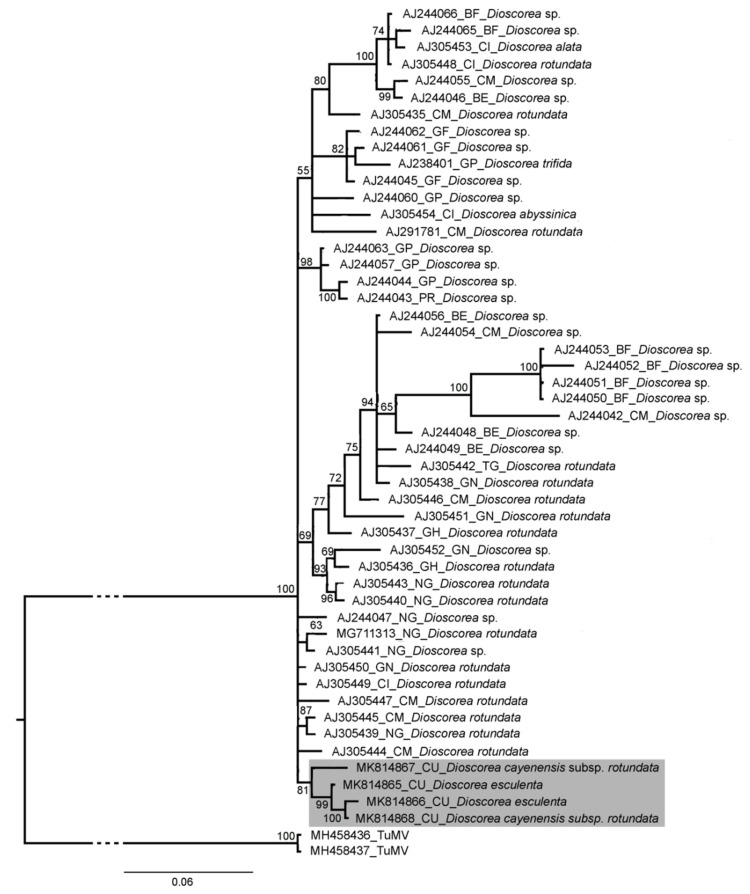
Consensus dendrogram obtained from Bayesian inference of the partial sequence (586 bp) of the capsid protein from Cuban isolates of yam mosaic virus and others in the GenBank database. The length of the arms and their significance are indicated at the nodes for a posterior probability greater than 0.5. The substitution model chosen was GTR+I+G4. For each isolate, the GenBank accession number, the geographical origin, and the *Dioscorea* species from which it was isolated are given. Turnip mosaic virus (TuMV) was used as an outgroup sequence for comparison. The scale bar indicates the number of nucleotide substitutions per site. (BF) Burkina Faso; (BJ) Benin; (CI) Ivory Coast; (CM) Cameroon; (CU) Cuba; (GF) French Guyana; (GH) Ghana; (GN) Guinea; (GP) Guadeloupe; (NG) Nigeria; (PR): Puerto Rico; (TG) Togo.

**Figure 5:**
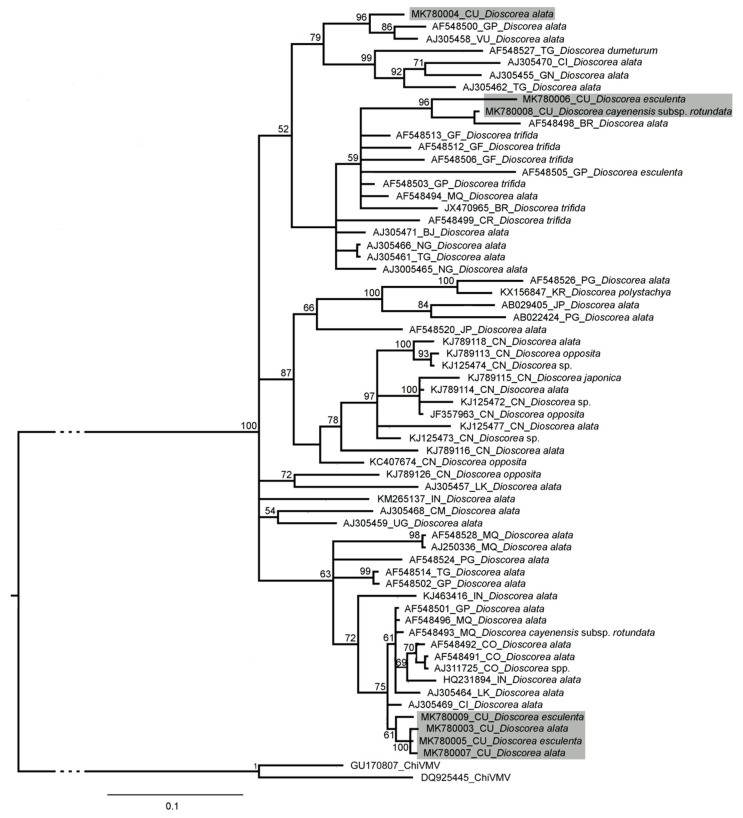
Consensus dendrogram obtained by Bayesian inference of the partial sequence (249 bp) of the capsid protein of Cuban isolates of yam mil mosaic virus and others in the GenBank database. The length of the arms and their significance are indicated at the nodes for a posterior probability greater than 0.5. The substitution model chosen was GTR+G4. For each isolate, the GenBank accession number, the geographical origin, and the *Dioscorea* species from which it was isolated are indicated. Chilli vein mottle virus (ChiVMV) was used as an outgroup sequence for comparison. The scale bar indicates the number of nucleotide substitutions per site. (BF) Burkina Faso; (BJ) Benin; (BR) Brazil; (CI) Ivory Coast; (CM) Cameroon; (CN) China; (CO) Colombia; (CR) Costa Rica; (CU) Cuba; (GF) French Guyana; (GN) Guinea; (GP) Guadeloupe; (IN) India; (JP) Japan; (KR) South Korea; (LK) Sri Lanka; (MQ) Martinique; (NG) Nigeria; (PG) Papua New Guinea; (TG) Togo; (VU) Vanuatu.

The partial sequences of the samples positive for the presence of YMV (586 bp) and YMMV (249 bp) showed high percentages of similarity to both viruses by homology search in the GenBank database. Three of the six YMMV sequences obtained were identical, so only four of them were assigned GenBank accession numbers (MK814865–MK814868), while the seven YMMV sequences were different (MK780003–MK780009). Bayesian analysis of these sequences and a selection of sequences corresponding to these viruses from the GenBank database revealed several clusters (Figure 4 and Figure 5).

### 2.3. Severity of Potyvirus-Associated Diseases

The results of applying the severity scale to four cultivars of the Cuban germplasm collection of *Dioscorea* spp. are shown in Table 4. Similar disease severity scores were obtained within each of the cultivars evaluated in the two growing seasons, except in the second season for the cultivar “Guinea” and the cultivar “Cush-cush”. In all cases, the ACP-ELISA assay confirmed the presence of potyvirus in symptomatic plants tested.

The analysis of the mean values of the severity scale allowed the determination of the highest values in the second evaluation in all cultivars. When comparing this indicator at this time of evaluation, three groups could be distinguished. “Cush-cush” and “Volador” cultivars with the highest and lowest values on the severity scale, respectively, and “Belep” and “Guinea” cultivars with intermediate values.

In the cultivar “Volador” (*D. bulbifera*), no symptoms were observed during the two evaluation seasons, nor was the presence of potyvirus detected by the ACP-ELISA test. In 77.6% of the evaluations carried out, asymptomatic plants (grade 1) were found in this cultivar. For the cultivars “Belep” (*D. alata*) and “Guinea” (*D. cayenensis* subsp. *rotundata*), severity scores ranged from 1 to 3, corresponding to less than 50% of the total number of leaves with symptoms. In “Cush-cush” (*D. trifida*), the mean disease severity was 4.75, with the SM-t symptom being the most prominent. In the second season, a mean disease severity of 5 was reached from 90 dap, which differs from the rest of the cultivars evaluated.

The application of the severity scale allowed differentiating the responses to potyvirus-associated diseases among cultivars of *Dioscorea* spp. at INIVIT’s germplasm collection, which is of great importance for the management of associated diseases.

### 2.4. Incidence of Potyvirus-Associated Diseases

The results of the symptoms classification of the 2016–2017 growing season, as well as the ACP-ELISAs performed on the asymptomatic samples, are presented in Table 5. A total of 160 leaf samples were collected in the Cooperativa de Crédito y Servicio (CCS) “Fidel Claro” and 180 in the CCS “Juan Verdecia” of the cultivar “Guinea”, while 200 leaf samples of the cultivar “Belep” were collected in the CCS “José Antonio Echeverría”. The results of the following two seasons were statistically similar.

As in the sampling carried out in the areas of the Cuban germplasm collection of *Dioscorea* spp., SM-r, and EC-r symptoms were identified in *D. cayenensis* subsp. *rotundata* and YB-a and MM-a symptoms in *D. alata*. However, the presence of MM-r or GSZ-a symptoms was not observed in the two cultivars evaluated. Neither CE nor LD symptoms were observed (Table 1). In the cultivar “Guinea” the most characteristic symptom was CC-r, and in the cultivar “Belep” the most characteristic symptom was YB-a.

All symptomatic samples analyzed by ACP-ELISA were positive, similar to the results obtained in the *Dioscorea* spp. germplasm collection. However, 15.6% (27/173) of the asymptomatic samples collected were positive by ACP-ELISA.

The incidence of diseases caused by potyviruses in the production areas of the municipality of Camajuaní varied between 64% and 78%. The high incidence values found in the production areas of the two main *Dioscorea* species grown in Cuba demonstrate the need to establish disease management programs in yam cultivation.

## 3. Discussion

The most common symptoms observed on the leaves of *Dioscorea* spp. plants at INIVIT’s germplasm collection have been identified; among them are SM, CC, YB, MM, GSZ, SC, and LD. In this sense, Eni et al. [14] only observed SM symptom associated with *D. rotundata* in their evaluation of 628 foliar samples from grower farms in Ghana, but not in *D. alata* and *D. cayenensis* species in production areas in Benin. Toualy et al. [15] also reported the highest incidence of this symptom in cultivars of the *D. cayenensis-rotundata* complex in plantations in Bringakro province, Ivory Coast. Furthermore, MM symptom was reported as the most frequently associated with viral diseases in *Dioscorea* spp.

The predominance of one type of symptom in some species, i.e., SM in *D. trifida* and GSZ in *D. esculenta*, is consistent with those reported by Filho et al. [16], who found more than 90% incidence of the SM symptom when evaluating cultivars of this species in northeastern Brazil, as well as Vijayanandraj et al. [17], who detected the occurrence of GSZ symptom in more than 40% of *D. esculenta* plants in India.

In the specific case of the SM symptom reaching the entire leaf in *D. trifida* cultivars, similar results were reported by Seal et al. [6] and Mambole et al. [18], who described this symptom in practically 100% of leaves in commercial cultivars of *D. trifida* in the West Indies.

Symptom manifestation in plants is the result of the interaction of at least four factors: the pathogen [19,20], the host [21], possible interaction with other viruses [22], and the environment [23]. Symptoms may or may not manifest themselves [24,25] without their absence being a definitive criterion for determining the phytosanitary status of plants. Therefore, the need for serial, plant-by-plant assessments of symptomatology from the earliest stages of plant development in the field is very useful for obtaining more reliable results. In *Dioscorea*, it remains to be investigated the possible effect of mixed infections on plant symptomatology and potyvirus fitness. Therefore, even knowing the viral combinations present, it would be necessary to implement efficient inoculation systems and the construction of infectious clones to elucidate these specific responses.

In the absence of other methods to obtain virus-free propagating material, growers can establish a positive selection system by regularly observing the symptoms present in their plantations and marking plants that do not show symptoms throughout the cycle.

Positive ELISA reactions were obtained in all *Dioscorea* spp. samples tested, except those with SC and LD symptoms. These symptoms were also not associated with the presence of potyvirus in *Dioscorea* spp. in studies conducted in the Southeast Pacific [26], sub-Saharan Africa [14], India [17], and South America [16]. The fragment obtained by RT-PCR was consistent with that described by Zheng et al. [27], confirming the presence of potyviruses associated with the symptom-species groups analyzed.

Wulandari and Ermayanti [28] reported 100% agreement of results between ACP-ELISA and RT-PCR methods in a certification program of *Dioscorea* spp. However, the difference between the ACP-ELISA and RT-PCR results in the specific case of BA-b and ML-b could be due to the presence of another potyvirus not recognized by the generic primers used, as previously informed by Dey et al. [29] in *D. bulbifera* in Florida, USA.

The results obtained using the primer pairs for YMV and YMMV proposed by Mumford and Seal [30] confirm the presence of these viruses in *Dioscorea* spp. samples in six and seven symptom-species groups, respectively (Table 3). All samples positive for YMV or YMMV by RT-PCR were also classified as potyvirus positive by ACP-ELISA. This confirms the potential of this immunoassay as a starting point for potyvirus-free seed certification programs in commercial cultivars of *D. alata* or *D. cayenesis* subsp. *rotundata*.

Neither YMV nor YMMV was detected in the samples of *D. bulbifera* during three years of symptomatic leaf collection and diagnosis. In this regard, none of these potyviruses were found in cultivars of this species in studies conducted in the Southeast Pacific [31], sub-Saharan Africa [14,32], and India [28]. This might indicate the presence of dominant innate or atypical resistance to YMV and YMV isolates circulating in cultivars of this species at INIVIT’s germplasm collection.

The detection of mixed infections in three of the described symptom-species groups (YB-a, MM-a, SM-r) is consistent with other results reported in *D. alata* and *D. cayanensis* subsp. *rotundata*, when even a third virus was found [14,33,34].

Through the detection and characterization of two potyviruses in more than 120 cultivars of the five *Dioscorea* spp. species from Africa, Asia, Oceania, and America conserved at INIVIT’s germplasm collection, a gradient of interactions between plants and these viruses has been established. In this sense, the similarity or difference in origin between plants and pathogens has contributed to obtaining such diverse patterns of plant-microorganism interactions, consistent with what has been pointed out by Elena et al. [35] and Escriu [36].

The Cuban cultivars of *D. trifida*, a species native to the Americas [1] evaluated in this research, do not complete their cycle, which could be related to YMV, of which an African origin has been proposed [37]. The difference in origin between virus and host is probably one of the causes of the high susceptibility shown by this species, as expansion to new hosts has been documented to cause increased virulence, especially for RNA viruses [38].

Under the experimental growing conditions, *D. alata* and *D. cayenensis* are both affected by YMV and YMMV, but the cultivars evaluated were able to complete the growing cycle. Bousalem et al. [37] proposed the African origin of YMV together with the “rotundata-cayenesis complex”, as well as the common origin of YMMV and *D. alata* in Southeast Asia. The common origin may have facilitated the co-evolution of viruses and hosts, thus establishing a balance between them [39,40]. For this reason, it has been suggested that viruses, as a “strategy”, avoid the destruction of their host [41,42] due to their complete dependence on the host cellular mechanism to complete their cycle (biotrophic).

The Cuban YMV isolates form a completely different group from the other YMV isolates evaluated, suggesting a single introduction into the country. On the other hand, the Cuban YMMV isolates were grouped in three clusters, suggesting multiple introductions to the country. These introductions of plant material may be related to the exchanges that often take place with germplasm banks of institutions from other regions.

The results presented for the cultivar “Volador” (*D. bulbifera*) are consistent with those reported for cultivars of this species in evaluations conducted in some production areas in Cameroon [43]. For cultivars “Belep” and “Guinea”, the results agree with those reported by Odu et al. [44], who found mean disease severity scores lower than 3. Then, the disease severity results obtained for cultivars “Belep” and “Guinea” suggest that they are tolerant to potyvirus isolates present in the *Dioscorea* spp. germplasm collection in Cuba. In addition, the average disease severity found in the cultivar “Cush-cush” (*D. trifida*) allows it to be classified as highly susceptible to potyvirus, which is the case according to Azeteh et al. [43] and Mignouna et al. [45].

The application of the severity scale allowed differentiation of responses to potyvirus-associated diseases among cultivars of *Dioscorea* spp. at INIVIT’s germplasm collection, which is of great importance for disease management. Therefore, it is important to determine other epiphytological elements in the production areas of *Dioscorea* spp.

The most characteristic symptoms in cultivar “Guinea” and cultivar “Belep” were consistent under commercial farm conditions and in the *Dioscorea* spp. INIVIT’s germplasm collection. The number of asymptomatic samples that subsequently tested positive for ELISA was similar to that reported by Njukeng et al. [46], which suggests an attenuation of symptoms throughout the crop cycle. This fact emphasizes the need for serial sampling of symptomatology from the early stages of crop development for possible healthy donors.

In general, the incidence and severity of potyvirus-associated diseases in *Dioscorea* spp. plantations depend on the combination of several factors. Among these, alternative hosts found by potyviruses in weeds associated with the crop may be an important epiphytological element.

## 4. Materials and Methods

### 4.1. Symptom Classification

To determine the potyviruses present in the *Dioscorea* spp. at INIVIT’s germplasm collection, samples of symptomatic and asymptomatic leaves were collected from *D. alata*, *D. cayenensis*, *D. esculenta*, *D. bulbifera,* and *D. trifida*. The criterion for sampling with symptoms is based on all those leaves that, to the naked eye, are morphologically different from those described by Pérez-Camacho and Raz [47]. Sampling was carried out during the 2014–2015, 2015–2016, and 2016–2017 seasons, starting 30 dap of the crop, monthly, throughout the phenological development (6–12 months) of *Dioscorea* spp.

The collected samples were classified according to the type of symptom and species. The definition of the type of symptom induced by potyvirus was based on criteria used by researchers in several regions [14,15,16,26]. All observations and samples were collected at the same time of the year and in the early hours of the day between 90 and 120 dap. The sampled plants were marked for their description of symptom development. During the sampling period, the minimum and maximum temperature values were recorded, as well as rainfall and relative humidity, according to Agro-Meteorological Station 326 of the National Meteorological Network, located at INIVIT.

### 4.2. Potyvirus Detection

#### 4.2.1. Electron Microscopy

Leaf samples collected from symptomatic and asymptomatic groups were analyzed at Instituto de Patología Vegetal, Centro de Investigaciones Agropecuarias, Instituto Nacional de Tecnología Agropecuaria (IPAVE-CIAP-INTA), Argentina. The leaf dipping technique was used according to the procedure described by Truol et al. [48]. Dips were prepared from previously lyophilized samples and counterstained with 2% uranyl acetate and lead citrate (Sigma, St. Louis, MO, USA). The preparations were examined with a JEM 1200 EX II transmission electron microscope (JEOL, Tokyo, Japan).

#### 4.2.2. ELISA

An ACP-ELISA diagnostic system [49] using a validated monoclonal antibody for YMV, YMMV, and 71 other potyviruses (DSMZ, Braunschweig, Germany) [28], standardized according to the manufacturer’s instructions, with two replicates per sample, was used to detect potyviruses in the collected samples. The absorbance was determined at 405 nm on a Biotek® ELx-800 automated plate reader (BioTek Instruments, Inc., Winooski, VT, USA). The cut-off for the detection of potyvirus was calculated as twice the average absorbance value of the negative controls. All samples were analyzed individually.

#### 4.2.3. RT-PCR

For RT-PCR, total RNA was purified from 100 mg of leaves of *Dioscorea* spp., stored at −80 °C and positive for potyvirus by ACP-ELISA. The RNeasy® Plant Mini Kit (Qiagen, Hilden, Germany) was used according to the manufacturer’s recommendations. The concentration of extracted total RNA was determined from the optical density obtained at 260/280 nm in a BioPhotometer spectrophotometer (Eppendorf, Hamburg, Germany).

Complementary DNA (cDNA) was synthesized from 1.50 μg of total RNA using the High Capacity cDNA Reverse Transcription Kit (Applied Biosystems, Waltham, MA, USA) in a final volume of 20 μL. PCR reactions were performed in a final volume of 25 μL using 1 μL of cDNA, 1× commercial buffer, 0.2 mM of each dNTP, 0.4 μM of each primer (generic potyvirus, YMV, and YMMV) [27,30], and 1.25 U of Top Taq™ DNA Polymerase (Qiagen, Hilden, Germany) were used for this purpose. Primers NIb2F 5′-GTITGYGTIGAYGAYTTYAAYAA-3′ and NIb3R 5′-TCIACIACIGTIGAIGGYTGNCC-3′ amplify a fragment of approximately 350 bp of the *NIb* (viral replicase) gene; YMV forward 5′-ATCCGGGATGTGGACAATGA-3′ and reverse 5′-TGGTCCTCCGCCACATCAAA-3′ amplify a 586 bp fragment, and YMMV forward 5′-GGCACACATGCAAATGAAAGC-3′ and reverse 5′-CACCAGTAGAGTGAACATAG-3′ amplify a 249 bp fragment of the *CP* (capsid protein) gene and 3′ UTR region, respectively. PCR conditions included an initial 2 min cycle at 94 °C, followed by 35 cycles of denaturation at 94 °C for 30 s, hybridization at 55–58 °C for 45 s and extension at 72 °C for 1 min, and a final extension of 10 min at 72 °C. A Mastercycler® personal thermal cycler (Eppendorf, Hamburg, Germany) was used.

Amplification products were verified by 1.2% agarose gel electrophoresis in 1× TBE buffer (890 mM Tris-borate, 890 mM boric acid, and 20 mM EDTA). DNA fragments were stained with Midori Green Advanced DNA Stain (NIPPON Genetics EUROPE GmbH, Düren, Germany) and visualized under UV light [50].

#### 4.2.4. Sequencing and Analysis

Viral genome fragments were purified from amplicons of the expected size for YMV and YMMV using the QIAquick Gel Extraction Kit (Qiagen, Hilden, Germany). The purified products were then sequenced on an ABI Prism 3.730 xl sequencer (PE Applied Biosystems, Waltham, MA, USA), Macrogen Company (Seoul, Republic of Korea). The resulting sequences were edited using the SEQMAN program (DNASTAR). Consensus sequences were constructed and their identity was confirmed by comparison in the BLAST program (http://www.ncbi.nlm.nih.gov/BLAST/Blast.cgi, accessed on 18 May 2020). Multiple sequence alignments were performed using the MUSCLE algorithm [51] in the SeaView v. 4.4.2 program [52]. The substitution model was estimated using the program jModelTest v. 2.1.9 [53], according to the Akaike Information Criterion. The dendrograms were obtained by Bayesian inference in Mr Bayes 3.2.6 [54].

### 4.3. Severity of Potyvirus-Associated Diseases

Based on the knowledge of the symptoms associated with YMV and YMMV under natural infection conditions of *Dioscorea* spp. at INIVIT’s germplasm collection, and to evaluate the severity of the diseases associated with these viruses, the quantitative scale according to Azeteh et al. [44] and Mignouna et al. [46] was applied, based on the percentage of symptomatic leaves in the plants. Grade 1 of the scale corresponds to 0%, 2 to less than 25%, 3 between 26 and 50%, 4 between 51 and 75%, and 5 to more than 75% symptomatic leaves. For this purpose, 16 plants (4 groups of 4 plants) of the commercial cultivars “Guinea” (*D. cayenensis* subsp. *rotundata*) and “Belep” (*D. alata*), as well as the cultivars “Cush-cush” (*D. trifida*) and “Volador” (*D. bulbifera*) were evaluated. Three observations were made between 90 and 150 dap, at monthly intervals, during the 2015–2016 and 2016–2017 seasons (96 evaluations per cultivar). All samples were tested for the presence of potyvirus by ACP-ELISA. The average value of the severity scale grade was determined for each cultivar.

Statistical processing of the data obtained was carried out with the IBM SPSS Statistics, version 25 for Windows. In all cases, the assumptions of normality (Sapiro Wilk) and homogeneity of variance (Levene) were verified. A Friedman (*p* < 0.05) and Wilcoxon (*p* < 0.01) a posteriori analysis was performed to compare severity between the different evaluation times. In addition, a Kruskal–Wallis (*p* < 0.05) and Mann–Whitney U (*p* < 0.01) a posteriori analysis was performed to compare severity between cultivars.

### 4.4. Incidence of Potyvirus-Associated Diseases

To determine the incidence of potyvirus-associated diseases in the production areas, plantations of commercial *Dioscorea* cultivar “Guinea” were sampled in the CCS “Fidel Claro” and “Juan Verdecia”, while samples of *Dioscorea* cultivar “Belep” were collected in the CCS “José Antonio Echeverría” (Supplementary Material Figure S1), belonging to the municipality of Camajuaní, the largest planted area in the central region (more than 170 ha per year) and is among the first three in the country [11].

Sampling was carried out between 90 and 120 dap during the 2016–2017, 2017–2108, and 2018–2019 seasons. Zig-zag walks were performed over the diagonals [55], and every third group of 4 plants, symptomatic or asymptomatic, was collected randomly [48], for a total of 180 plants ha^−1^. Symptoms were classified as described above. For asymptomatic plants, leaves were collected from the middle part of the vines [55,56]. All collected samples were transported to the laboratory for analysis. All asymptomatic samples and 10% of the samples from each of the symptomatic groups were tested for the presence of potyvirus by ACP-ELISA, as previously described.

## 5. Conclusions

The symptomatology of *Dioscorea* spp. at INIVIT’s germplasm collection was described. Moreover, it was possible to detect the presence of YMV and YMMV, even in mixed infections. The classification of the cultivars “Belep” (*D. alata*) and “Guinea” (*D. cayenensis* subsp. *rotundata*) as tolerant to diseases associated with these viruses can be a starting point for establishing the management of diseases associated with potyviruses under production conditions, where it was found to have a high disease incidence. All this confirms the need for the establishment of certification programs for propagating material of *Dioscorea* spp.

## Figures and Tables

**Table 1:** Distribution of symptoms present in leaf samples collected from the different *Dioscorea* spp. at INIVIT’s germplasm collection.

Species	Total of Samples	Symptom						
		SM	CC	YB	MM	GSZ	SC	LD
*D. alata*	225			106	62	5	20	32
*D. cayenensis* subsp. *rotundata*	126	18	69		11		9	19
*D. trifida*	19	15			1		3	
*D. esculenta*	28					19	5	4
*D. cayenensis* subsp. *cayenensis*	53	1		22	17		5	8
*D. bulbifera*	28			1	1		23	3

(SM) severe mosaic; (CC) curling and chlorosis; (YB) yellow banding; (MM) mild mosaic; (GSZ) green swelling zones; (SC) severe chlorosis; (LD) leaf distortion.

**Table 2:** ACP-ELISA results in the described symptom-species groups.

Dioscorea spp.	Symptoms						
	SM	CC	YB	MM	GSZ	SC	LD
*D. alata*			(++)	(++)	(+)	(−)	(−)
*D. cayenensis* subsp. *rotundata*	(+)	(++)		(+)		(−)	(−)
*D. trifida*	(+++)			(+)		(−)	
*D. esculenta*					(+)	(−)	(−)
*D. cayenensis* subsp. *cayenensis*	(+)		(+)	(+)		(−)	(−)
*D. bulbifera*			(+)	(+)		(−)	(−)

(−) absorbance value (AV) less than the cut-off limit; (+) AV greater than the cut-off limit; (++) AV twice the cut-off limit; (+++) VA four times the cut-off limit. (SM) severe mosaic; (CC) curling and chlorosis; (YB) yellow banding; (MM) mild mosaic; (GSZ) green swelling zones; (SC) severe chlorosis; (LD) leaf distortion.

**Table 3:** Detection of yam mosaic virus (YMV) and yam mild mosaic virus (YMMV) by RT-PCR.

Virus	Symptom-Species Groups													
	YB-a	YB-b	GSZ-a	MM-t	MM-b	SM-c	MM-r	YB-c	MM-c	CC-r	SM-t	MM-a	SM-r	GSZ-e
Potyvirus	+	−	+	+	−	+	+	+	+	+	+	+	+	+
YMV	+	−	−	−	−	−	−	+	−	+	+	+	+	−
YMMV	+	−	−	−	−	+	+	−	+	−	−	+	+	+

(SM) severe mosaic; (CC) curling and chlorosis; (YB) yellow banding; (MM) mild mosaic; (GSZ) green swelling zones. (-a) *D. alata*; (-b) *D. bulbifera*; (-t) *D. trifida*; (-c) *D. cayenensis* subsp. *cayenensis*; (-r) *D. cayenensis* subsp. *rotundata*; (-e) *D. esculenta*. (*+*) positive sample; (−) negative sample.

**Table 4:** Disease severity caused by potyvirus in four cultivars of four *Dioscorea* species at INIVIT’s germplasm collection.

Cultivar	Season	Severity (Scale 1–5)				
		Range	1st Evaluation	2nd Evaluation	3th Evaluation	2nd Evaluation Media ± SE
“Belep”	2015–2016	1–3	1.63	2.13	2.00	2.06 ± 0.14 B
	2016–2017		2.00	2.00	1.88	
“Guinea”	2015–2016	2–3	2.50	2.50	2.13	2.44 ± 0.13 B
	2016–2017		2.75 a	2.38 ab	2.25 b	
“Volador”	2015–2016	-	1.00	1.00	1.00	1.00 ± 0.0 C
	2016–2017		1.00	1.00	1.00	
“Cush-cush”	2015–2016	4–5	4.25 b	4.75 ab	5.00 a	4.63 ± 0.16 A
	2016–2017		4.25 b	4.50 ab	5.00 a	

Scale grades: (1) no symptoms; (2) <25% of symptomatic leaves; (3) between 25% and 50%; (4) between 50% and 75%; (5) >75%. Different lowercase letters in the same row indicate that severity means are statistically different according to Friedman (*p* < 0.05) and Wilcoxon (*p* < 0.01) a posteriori analyses. Different capital letters indicate that severity means are statistically different according to Kruskal–Wallis (*p* < 0.05) and Mann–Whitney U (*p* < 0.01) a posteriori analyses. (SE) Standard error.

**Table 5:** Incidence of potyvirus-associated diseases in production areas, 2016–2017 season, of *Dioscorea* spp. in Camajuaní municipality, Cuba.

Cooperativas de Créditos y Servicios	Symptom					Asymptomatic		Incidence (%)
	SM-r	CC-r	YB-a	MM-a	ELISA (+)	ELISA (+)	ELISA (−)	
“Juan Verdecia”	31	73	-	-	104	12	64	64.4
“Fidel Claro”	39	77	-	-	116	6	38	76.3
“José A. Echeverría”	-	-	90	57	147	9	44	78

(-r) *D. cayenensis* subsp. *rotundata* cultivar “Guinea”; (-a) *D. alata* cultivar “Belep”. (SM) severe mosaic; (CC) curling and chlorosis; (YB) yellow banding; (MM) mild mosaic. ELISA (±): Plants diagnosed as ± for the presence of potyvirus according to an ELISA test.

## Data Availability

All data supporting the results and conclusions of the study are contained within the article. Generated sequences have been submitted to GenBank.

## Supplementary Materials

The following supporting information can be downloaded at: https://www.mdpi.com/article/10.3390/plants13182597/s1, Table S1: Monthly mean values of maximum, mean, and minimum daily temperatures and relative humidity recorded during the period of *Dioscorea* spp. sample collection; Figure S1: Location of the production units where the incidence of diseases caused by potyvirus in the *Dioscorea* spp. crop was determined. (A) CCS “Juan Verdecia”; (B) CCS “Fidel Claro”; (C) CCS “José Antonio Echevería”.
