# Supplementary material for: Occurrence of Yam Mosaic Virus and Yam Mild Mosaic Virus on Dioscorea spp. Germplasm Collection in Cuba—Epidemiology of Associated Diseases
Source: Plants (Basel). 2024 Sep 17;13(18):2597. doi: 10.3390/plants13182597 (PMC11435102; doi:10.3390/plants13182597)
Supplement: Supplementary file 1 [file plants-13-02597-s001.zip › Supplementary Material_Figure S1.pdf]

Figure S1. Location of the production units where the incidence of diseases caused by potyvirus in the *Dioscorea* spp. crop was determined. A) CCS "Juan Verdecia"; B) CCS "Fidel Claro"; C) CCS "José Antonio Echevería".

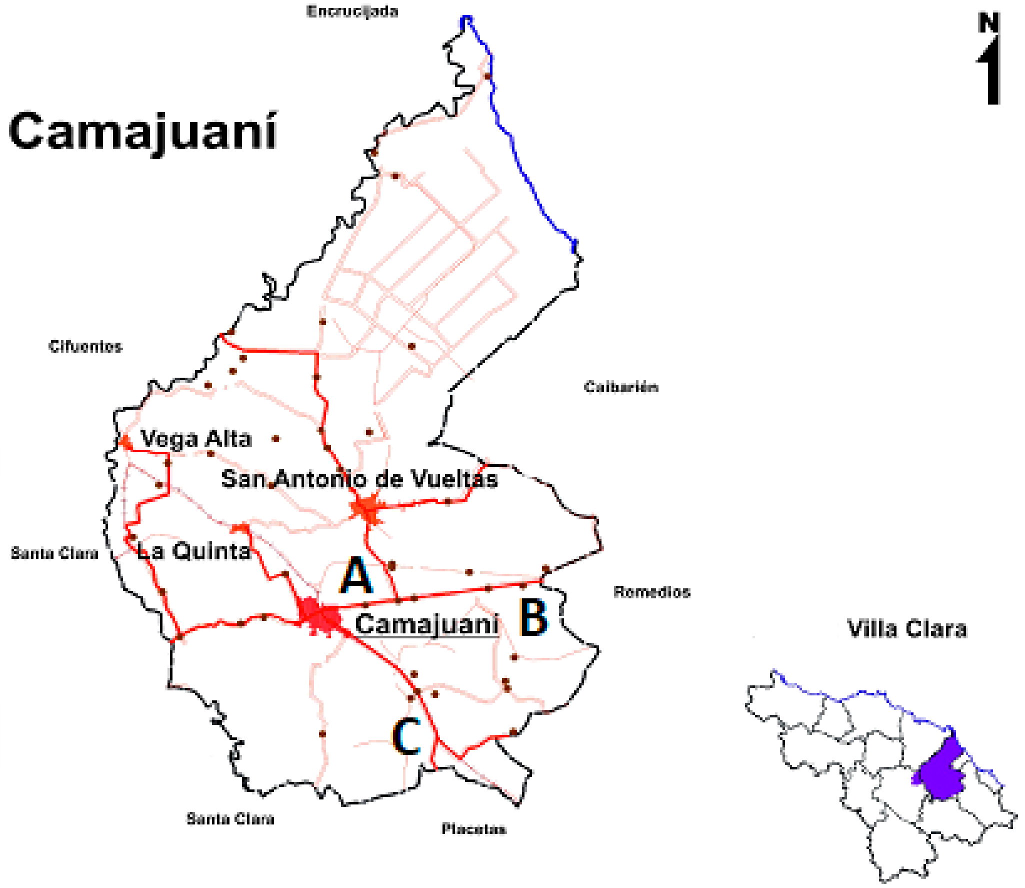

(CCS) Cooperativas de Créditos y Servicios.
