# Supplementary material for: Occurrence of Yam Mosaic Virus and Yam Mild Mosaic Virus on Dioscorea spp. Germplasm Collection in Cuba—Epidemiology of Associated Diseases
Source: Plants (Basel). 2024 Sep 17;13(18):2597. doi: 10.3390/plants13182597 (PMC11435102; doi:10.3390/plants13182597)
Supplement: Supplementary file 1 [file plants-13-02597-s001.zip › Supplementary Material_Table S1.pdf]

Table S1. Monthly mean values of maximum, mean and minimum daily temperatures and relative humidity recorded during the period of *Dioscorea* spp. sample collection.

Year	Temperature and relative humidity	Months											
		Jan	Feb	Mar	Apr	May	Jun	Jul	Aug	Sept	Oct	Nov	Dic
2015	TMax (°C)	27.9	27.8	31.5	33.3	31.5	32.7	34.3	33.6	33.1	31.1	30	30
	TMean (°C)	21.1	20.4	23.9	25.7	25.2	26.3	27	26.3	26.3	25	24.2	24.1
	TMin (°C)	15.5	13.5	17.3	19.3	19.8	21.4	21.4	21.5	22.1	20.7	19.9	19.9
	HR (%)	80	74	69	72	76	79	75	81	83	84	84	85
2016	TMax (°C)	26.7	26.7	30.4	30.9	32.1	32.6	33.2	32.6	32.7	30	28	29.7
	TMean (°C)	20.9	20.1	23.5	24	25.5	26.5	27.2	26.9	26.5	24.8	21.7	23.3
	TMin (°C)	15.9	14.1	17.5	18	20.1	22.3	22.7	22.8	22.3	21.1	16.3	18.2
	HR (%)	83	77	73	72	75	79	78	80	82	84	80	81
2017	TMax (°C)	28.4	29.4	29.2	31.3	32.1	32.8	33.4	33.6	31.7	29.9	28.1	26.8
	TMean (°C)	21.6	22.2	22.1	24.5	25.7	26.8	27	27	26.3	24.9	23.8	21.3
	TMin (°C)	15.6	15.9	15.6	18.9	20.7	22.7	22.4	22.5	22.6	21.3	18.1	16.6
	HR (%)	77	79	69	71	74	77	78	80	85	85	85	84
2018	TMax (°C)	25	28.8	28.8	31.6	29.7	32.3	33.8	33.2	32.7	31.6	30.7	28.4
	TMean (°C)	20.7	23.2	21.8	24.7	24.7	26.5	27.5	27.1	26.6	25.8	24.3	22.8
	TMin (°C)	17	18.1	15.4	19.2	21.7	22.3	22.7	22.6	22.6	21.5	19.2	18
	HR (%)	84	82	74	77	87	82	78	80	83	82	81	80

(Jan) January; (Feb) February; (Mar) March; (Apr) April; (May) May; (Jun) June; (Jul) July; (Aug) August; (Sept) September; (Oct) October; (Nov) November; (Dec) December.
(TMax) maximum temperature; (TMean) Mean temperature; (TMin) minimum temperature.
